# Carfilzomib/pomalidomide single-agent or in combination with other agents for the management of relapsed/refractory multiple myeloma: a meta-analysis of 37 trials

**DOI:** 10.18632/oncotarget.10768

**Published:** 2016-07-21

**Authors:** Yandun Zou, Xiaoyan Ma, Haiying Yu, Chunling Hu, Limei Fan, Xuehong Ran

**Affiliations:** ^1^ Internal Medicine, Guang Dong Women and Children Hospital, Guang Zhou, China; ^2^ Department of I.C.U, Weifang People's Hospital, Weifang, China; ^3^ Department of Pediatrics, Weifang People's Hospital, Weifang, China; ^4^ Department of Hematology, Weifang People's Hospital, Weifang, China

**Keywords:** pomalidomide, carfilzomib, lenalidomide, bortezomib, multiple myeloma

## Abstract

**Purpose:**

The use of carfilzomib/pomalidomide single-agent or in combination with other agents in patients with refractory/relapsed multiple myeloma (RRMM) was not clearly clarified in clinical practice. We sought to compile the available clinical reports to better understand the efficacy and safety of carfilzomib (CFZ) and pomalidomide (POM).

**Results:**

Based on our research criteria, we identified 37 prospective studies that evaluated 1160 patients. Analysis of subgroup differences between carfilzomib single-agent and CFZ/DEX dual combination showed significantly(*P* < 0.001, I^2^ = 96.3%), suggesting the overall response rate (ORR) of 66% attained from CFZ/DEX dual combination seemed to be higher than that of 28% from carfilzomib single-agent. And, the same trend favoring CFZ/DEX dual combination was found in ≥VGPR and CBR analysis. The ORR of 31% attained from POM/DEX dual combination was superior to that of 19% from pomalidomide single-agent(*P* < 0.001, I^2^ = 94.4%). And, the same trend favoring POM/DEX dual combination was found in ≥VGPR and CBR analysis. However, the ORR of 83% attained from POM/BOR/DEX triplet combination was superior to that of 31% from POM/DEX dual combination(*P* < 0.001, I^2^ = 99.1%). And, the same trend favoring POM/BOR/DEX triplet combination was found in ≥VGPR analysis.

**Methods:**

We searched published reports including carfilzomib and (or) pomalidomide therapy for RRMM who had received bortezomib and (or) lenalidomide.

**Conclusion:**

Pomalidomide/Carfilzomib plus dexamethasone seemed to attain a superior response rate compared with pomalidomide/carfilzomib single-agent. Furthermore, the combination of pomalidomide, bortezomib and dexamethasone resulted in a much higher response rate compared with pomalidomide plus dexamethasone regimen. These results needed more validation in future trials.

## INTRODUCTION

In the past decades, the administration of novel agents (thalidomide lenalidomide and bortezomib) had produced a pronounced shift in the treatment framework for myeloma patients. And, treatment options and corresponding patient outcomes had greatly improved because of them. However, myeloma still remained incurable, and most patients would ultimately relapse and resist these agents [1]. Relapsed disease was characterized by increasingly lower remission rate even following salvage therapy. And, survival among those in whom lenalidomide, bortezomib, and thalidomide have failed was especially poor [2]. So, there was still an urgent need for new treatments to improve outcomes for these patients with RRMM.

Pomalidomide was a potent immunomodulatory drug that was FDA-approved for treatment of patients with RRMM. Preclinical studies had shown pomalidomide had robust antiangiogenic, antiapoptotic, and tumor necrosis factor-a inhibitory activity, stimulating antibody-dependent cytotoxic T-cell activity. Pomalidomide single-agent had been found to be active in relapsed/refractory patients [3, 4]. Preclinical studies had shown pomalidomide plus dexamethasone (DEX) had synergistic antiproliferative effects in LEN-resistant myeloma cells [5]. This pomalidomide dual regimen (POM/DEX) had been found to be active in several clinical trials with participants with RRMM [4, 6–13]. Meanwhile, several trials had shown pomalidomide triplet combinations (CFZ-POM-DEX, BOR-POM-DEX, CYC-POM-DEX et al.) also were effective for patients with RRMM [13–23].

Meanwhile, several trials had demonstrated the activity of carfilzomib single, dual and triplet combination regimens [17, 18, 24–41]. However, which strategy would be the optimal therapy for patients with RRMM still remains undefined. Furthermore, these published reports consisted of the clinical trials with small sample sizes, and these small trials were not enough power to determine the efficacy and safety of pomalidomide and carfilzomib. The present pooled analysis highlighted the two most recently approved anti-MM agents, carfilzomib and pomalidomide, and looked ahead toward optimal regimens in patients with RRMM.

## RESULTS

### Characteristics of the published reports of carfilzomib and pomalidomide

Based on our research criteria, we identified 37 prospective studies of carfilzomib and pomalidomide enrolling a total of 3432 patients with RRMM [13–21]. Of them, nine evaluated outcomes from carfilzomib single-agent [24–32]; eleven evaluated outcomes from carfilzomib combination regimens [17, 18, 31, 33–41]; two evaluated outcomes from pomalidomide single-agent [3, 4]; sixteen evaluated outcomes from pomalidomide combination regimens [4, 6–23].The characteristics of these trials were shown in Table 1.

**Table 1:** Characteristics of included studies

Author, year Strategy		Age (M)	F/M (n/N)	TFD (Y) (M)	Cytogenetic F/U/M	Pom/CFZ schedule	Prior therapy (M)	Prior therapy		Regimen	ORR	PFS (m)	OS(m)
								Bort	Lena				
Pomalidomide Trials													
Richardson 2014 [4]		61	55/53		43/30/35	21/28(4mg)	5 (1-12)	76	86	Poma alone	21%	9.5	30
Richardson 2013-1 [3]		66	20/18	5.5	-	21/28(4mg)	6 (2-17)	28	31	Poma alone	18%	4.6	18.3
Miguel 2013 [6]		64	121/181	5.3	—	21/28(4mg)	5 (2-14)	302	302	Pom+LoDex	32%	4.0	12.7
Lacy 2011 [7]		62	8/27	5.2	15/−/−	28/28(2mg)	-	35	35	Pom+LoDex	26%	6.5	78%(6m)
		61	14/21	6.0	21/−/−	28/28(4mg)	-	35	35	Pom+LoDex	29%	3.2	67%(6m)
Lacy 2010 [8]		61.5	11/23	5.2	14/20/−	28/28(2mg)	-	20	34	Pom+LoDex	32%	4.8	13.9
Richardson 2014 [4]		64	58/55	-	31/56/26	21/28(4mg	5 (1-13)	113	113	Pom+LoDex	33%	4.2	16.5
Leleu 2013 [9]		60	—	5.1	8/15/10	21/28(4mg)	5(1-13)	34	36	Pom+LoDex	34%	5.4	14.9
		60	—	6.5	13/19/9	28/28(4mg)	5(2-10)	34	39	Pom+LoDex		3.7	14.8
Lacy 2009 [10]		65.5	24/36	3.6	19/38/2	28/28(2mg)	-	20	21	Pom+LoDex	63%	11.6	76%(2y)
Leleu 2015 [11]		63	20/30	3	60/0/0	21/28(4mg)	3 (1-10)	48	50	Pom+LoDex	22%	2.8	12
Matsue 2015[12]		68	5/7	5.15	-	21/28(2mg,4mg)	6.0 (4-10)	12	12	Pom+LoDex	25%	5.5	
Baz 2016-B [13]		64	13/23	-	-	21/28(4mg)	4 (2-12)	28	-	Pom+LoDex	39%	4.4	16.8
Baz 2016-A [13]		69	3/7	-	-	21/28(4mg)	5 (4-12)	10	-	PCD	50%	-	-
Baz 2016-C [13]		65	16/18	-	-	21/28(4mg)	4 (2-9)	24	-	PCD	65%	9.5	NR
Shah 2015 5/14 [14]		57	-	-	-	21/28(4mg)	8(2-22)	-	-	OPD	50%	-	-
Shah 2015 2/7 [14]		66	-	-	-	-	4.5(2-11)	-	-	OPD	59%	-	-
Mark	2012 [15]	65	25/21	-	20/24	28/28(4mg)	5 (3–15)	42	46	ClaPD	61%	8.13	85%(9.4m)
Mark	2013 [16]	-	-	-	-	28/28(4mg)	5 (3-15)	90	97	ClaPD		8.1	-
Shah	2012/2015 [17,18]	63.5	12/20	5.0	18/6/5/1	21/28(4mg)	6 (1 -15)	31	32	CPD	64%	7.4	-
Shah	2013 [19]	64	27/45	5.1		21/28(4mg)	6 (2-15)	62	67	CPD		12	16.3
Larocca 2013 [20]		69	27/28	53	31/13/11	21/28(2.5mg)	3 (1-3)	46	55	PCP	51%	10.4	73%(14.8m)
Lacy 2014 [21]		66	24/23	49	4/38/5	21/28(4mg)	2 (1-5)	27	47	PVD	85%	10.7	-
Mikhael 2013 [22]		66	8/8	45	-	21/28(4mg)	3 (1-6)	8	16	PVD	83%	-	-
Richardson 2013-2 [23]		57	-	-	-	14/21(4mg)	2 (1-4)	21	21	PVD	75%	-	-
Carfilzomib Trials													
Vij 2012-1 [24]		63	17/18	3.6	25/9/1	20mg	3.0(1-13)	35	13	CFZ alone	17%	4.6m	29.9m
Vij 2012-2[25]		65	53/76	3.6	103/19/7	20/27mg	2 (1-4)	3	76	CFZ alone	48%	54.3%(9m)	-
Siegel 2012 [26]		63	111/155	5.4	159/75/32	20/27mg	5 (1-20)	265	249	CFZ alone	24%	3.7	15.6
Jagannath 2012[27]		63.5	21/25	5.5	33/7/5	20mg	5 (2-16)	46	42	CFZ alone	17%	3.5m	-
Jakubowiak 2013 [28]		65	76/91	5.6	-	20/27mg	5 (1-20)	166	-	CFZ alone	25%	4.6	19
		63	21/41	5.3	-	20/27mg	5 (2-12)	62	-	CFZ alone		3.5	9.3
Badros 2013 [29]		64	22/28	6.3	32/13/5	15/20/27mg	5 (1-15)	48	44	CFZ alone	25%	-	-
Hajek R 2015 [30]		65	11/22	4.7	22/7/4	20/36/45/56/70mg	5(1-9)	30	-	CFZ alone	19%	3.7	10.2
Papadopoulos 2015 [31]		63.3	75/82	-	-	20/27mg	-	-	-	CFZ alone	49%	7.0	-
Lendvai 2014[32]		63	25/19	-	23/20/1	20/56mg	5 (1-11)	44	-	CFZ alone	61%	4.1	20.3
Kaufman 2014[40]		64.5	-	-	-	20/36/45mg	-	-	-	CP	50%	14.3	-
Berdeja 2015 [33]		66	27/17	-	-	20/27/36/45mg	5 (1-10)	-	-	CP	67%	7.7	67%(24m)
Papadopoulos 2015[31]		59.5	5/17	3.6	14/7/1	20/36/45/56/70mg	4(2-9)	21	-	Cd	55%	6.2	-
Berenson 2014-2[38]		63	-	-	-	20/45/56/70/88mg	1(1-2)	-	-	Cd	67%	-	-
Dimopoulos 2015[39]		-	-	-	-	20/56mg	-	-	-	Cd	77%	18.7	-
Niesvizky 2013[35]		61.5	18/22	3.3	25/11/4	15/20/27mg	2 (1-3)	30	28	CRd	63%	10.2	-
Stewart 2015[36]		64	181/215	3.0	48/147/201	20/27mg	2(1-3)	261	79	CRd	87%	26.3	73.3%(2y)
Wang 2013[37]		61.5	36/48	3.1	57/22/5	20/27mg	2 (1-5)	65	59	CRd	69%	15.4	-
Shan2012/2015 [17,18]		64	12/20	5.9	10/−/−	20/27/36/45/56mg	6 (2-12)	31	32	CPD	50%	7.2	20.6
Vesole 2015 [41]		61	7/10	4	3/12/2	15/20/27mg	4 (1-9)	17	16	QUAD	53%	12	NR
Berenson 2014-1 [34]		67	13/25	4.2	-	20/27/36/45mg	-	-	-		43%	8.3	15.8

Abbreviations: F female; M male; (M) median; TFD time from diagnosis; F/U/M Favor/Unfavor/miss; Bor bortezomib; Lena lenalidomide; Pom pomalidomide;Dex Low dose dexamethasone; PCD pomalidomide cyclophosphamide and dexamethasone;OPD Oprozomib, Pomalidomide, and Dexamethasone; ClaPD Clarithromycin pomalidomide, and dexamethasone, CPD Carfilzomib, pomalidomide, and dexamethasone; PCP pomalidomide cyclophosphamide and prednisone; PVD pomalidomide bortezomib and dexamethasone; CP Carfilzomib, panobinostat; Cd Carfilzomib, dexamethasone; CRd Carfilzomib, lenalidomide, and dexamethasone; CPD Carfilzomib, pomalidomide, and dexamethasone; QUAD carfilzomib, lenalidomide, vorinostat and dexamethasone; Replacement of bortezomib with carfilzomib from bortezomib combination therapy; ORR overall response rate; NR: Not reach; 54.3%(9m) 54.3% progression free at 9 months;78%(6m) 78% alive at 6months;67%(6m) 67% alive at 6months; 76%(2y) 67% alive at 2 years; 85%(9.4m) 85% alive at 9.4months; 73%(14.8m) 73% alive at 14.8months; 67%(24m) 67% alive at 24months; 73.3%(2y) 73.3% alive at 2 years.

### Response rate to carfilzomib single-agent and carfilzomib combination regimens

**Figure 1:**
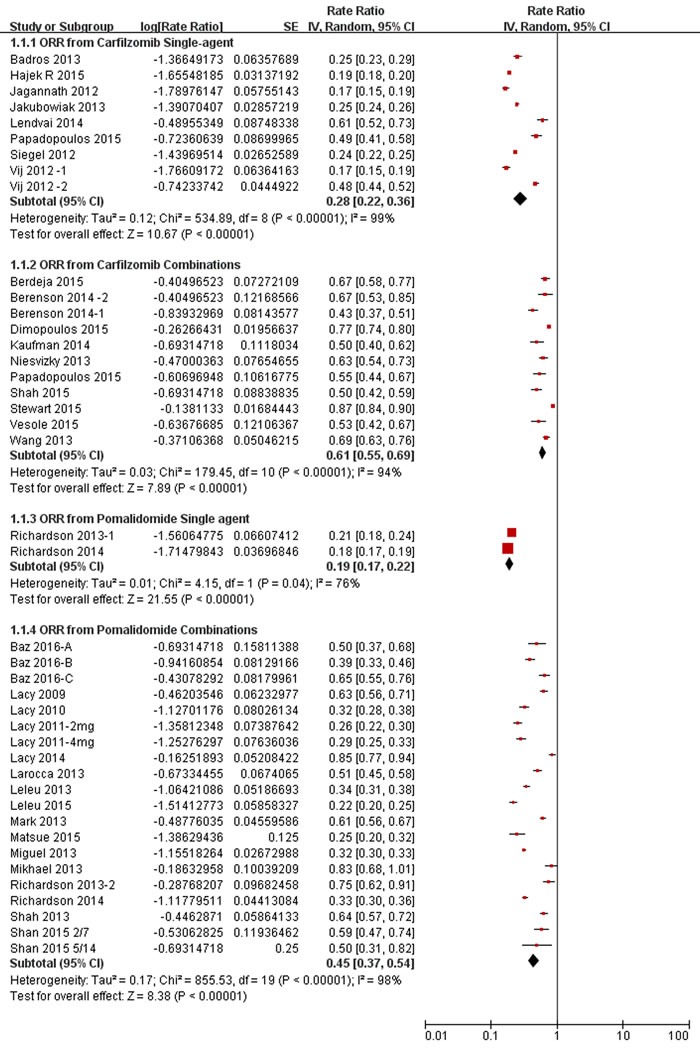
Meta-analysis of the overall response rate (ORR) of carfilzomib/pomalidomide single agent and combination regimens in patients with relapsed and refractory multiple myeloma n, number of the enrolled patients. CI, 95% confidence interval. Random, random-effects model.

Finally, nine trials enrolling a total of 957 patients evaluated the treatment effects on overall response of carfilzomib single-agent for the management of patients with RRMM. As shown in Figure 1, pooled analysis showed ORR was 28% of carfilzomib single-agent. Eleven trials enrolling a total of 1169 patients evaluated the treatment effects on overall response of carfilzomib combination regimens in patients with RRMM. Carfilzomib combination regimens resulted into an impressive ORR of 61% (Figure 1), which was higher than that of 28% from carfilzomib single-agent (*P* < 0.001, *I*^2^ = 97.1%) (Table 2).

**Table 2:** Summary of Response Outcomes from Carfilzomib and Pomalidomide

Outcomes	CFZ strategy	Num of Studies	RR (95% CI)	Test for subgroup heterogeneity	Outcomes	POM strategy	Num of Studies	RR (95% CI)	Test for subgroup heterogeneity
CFZ sing-agent versus CFZilzomib combination regimens					POM sing-agent versus POM combination regimens				
ORR	A:CFZ single-agent	9	0.28 [0.22,0.36]	A V BI^2^=97.1%,P<0.001	ORR	A:POM single-agent	2	0.19 [0.17,0.22]	A V BI^2^=97.9%,P<0.001
	B:CFZ combinations	11	0.61 [0.55,0.69]			B:POM combinations	16	0.45 [0.37, 0.54]	
≥VGPR	A:CFZ single-agent	4	0.10 [0.06,0.15]	A V BI^2^=95.6%,P<0.001	≥VGPR	A:POM single-agent	2	0.02[0.02,0.04]	A V BI^2^=97.2%,P<0.001
	B:CFZ combinations	9	0.34 [0.25,0.46]			B:POM combinations	15	0.15[0.10,0.24]	
CBR	A:CFZ single-agent	8	0.37 [0.31, 0.44]	A V BI^2^=97.8%,P<0.001	CBR	A:POM single-agent	2	0.36 [0.27, 0.48]	A V BI^2^=82.1%,P=0.02
	B:CFZ combinations	9	0.76 [0.69, 0.84]			B:POM combinations	12	0.54 [0.46, 0.64]	
SD	A:CFZ single-agent	7	0.31 [0.25, 0.39]	A V BI^2^=90.5%,P=0.001	SD	A:POM single-agent	1	0.48 [0.44, 0.53]	A V BI^2^=76.1%,P=0.04
	B:CFZ combinations	8	0.15 [0.11, 0.22]			B:POM combinations	13	0.28 [0.17, 0.47]	
**CFZ sing-agent versus dual combinations versus triplet combinations**					**POM sing-agent versus dual combinations versus triplet combinations**				
ORR	A:CFZ single-agent	9	0.28 [0.22,0.36]	A V B: I^2^=96.3%,P<0.001	ORR	A:POM single-agent	2	0.19 [0.17,0.22]	A V B:I^2^=95.1%,P<0.001
	B:CFZ + DEX	3	0.66 [0.53, 0.83]	B V C: I^2^=0%,P=0.57		B:POM + DEX	9	0.31 [0.26, 0.36]	B V C: I^2^=99.1%,P<0.001
	C:CFZ +LEN+ DEX	3	0.73 [0.59, 0.90]	B V D: I^2^=0%,P=0.77		C:POM+BOR+DEX	3	0.83 [0.76, 0.90]	B V D: I^2^=98.1%,P<0.001
	D:CFZ+POM+DEX	1	0.64 [0.57, 0.72]			D:POM+CFZ+DEX	1	0.64 [0.57, 0.72]	
≥VGPR	A:CFZ single-agent	4	0.10 [0.06,0.15]	A V B:I^2^=96.3%,P<0.001	≥VGPR	A:POM single-agent	2	0.02[0.02,0.04]	A V B:I^2^=92.7%,P<0.001
	B:CFZ + DEX	3	0.37 [0.23, 0.61]	B V C: I^2^=0%,P=0.51		B:POM + DEX	7	0.09 [0.05, 0.16]	B V C:I^2^=95.3%,P<0.001
	C:CFZ +LEN+ DEX	3	0.46 [0.29, 0.74]	B V D: I^2^=3.8%,P=0.31		C:POM+BOR +DEX	3	0.43 [0.30, 0.60]	B V D: I^2^=90.5%,P=0.001
	D:CFZ+POM+DEX	1	0.28 [0.25, 0.32]			D:POM+CFZ+DEX	1	0.28 [0.25, 0.32]	
CBR	A:CFZ single-agent	8	0.37 [0.31, 0.44]	A V B:I^2^=93.4%,P<0.001	CBR	A:POM single-agent	2	0.36 [0.27, 0.48]	A V B:I^2^=41.1%,P=0.19
	B:CFZ + DEX	2	0.75 [0.55, 1.01]	B V C: I^2^=0%,P=0.63		B:POM + DEX	6	0.44 [0.39, 0.50]	B V D: I^2^=98.6%,P<0.001
	C:CFZ +LEN+ DEX	3	0.81 [0.70, 0.94]	B V D: I^2^=0%,P=0.61		C:POM+BOR +DEX	-		
	D:CFZ+POM+DEX	1	0.81 [0.74, 0.89]			D:POM+CFZ+DEX	1	0.81 [0.74, 0.89]	
SDR	A:CFZ single-agent	7	0.31 [0.25, 0.39]	A V B:I^2^=0%,P=0.34	SDR	A:POM single-agent	1	0.48 [0.44, 0.53]	A V B:I^2^=82.7%,P=0.02
	B:CFZ + DEX	2	0.15 [0.03, 0.68]	B V C: I^2^=0%,P=0.86		B:POM + DEX	9	0.37 [0.30, 0.45]	
	C:CFZ +LEN+ DEX	3	0.13 [0.08, 0.21]			C:POM+BOR +DEX	-		
	D:CFZ+POM+DEX	-				D:POM+CFZ+DEX	-		

Abbreviations: CFZ carfilzomib; RR response rate; POM pomalidomide; ORR overall response rate (≥PR); ≥VGPR at least very good partial response; CBR clinical benefit rate; SDR stable disease rate; DEX dexamethasone; LEN lenalidomide; BOR bortezomib; A V B Test for subgroup heterogeneity between A and B; B V C Test for subgroup heterogeneity

**Figure 2:**
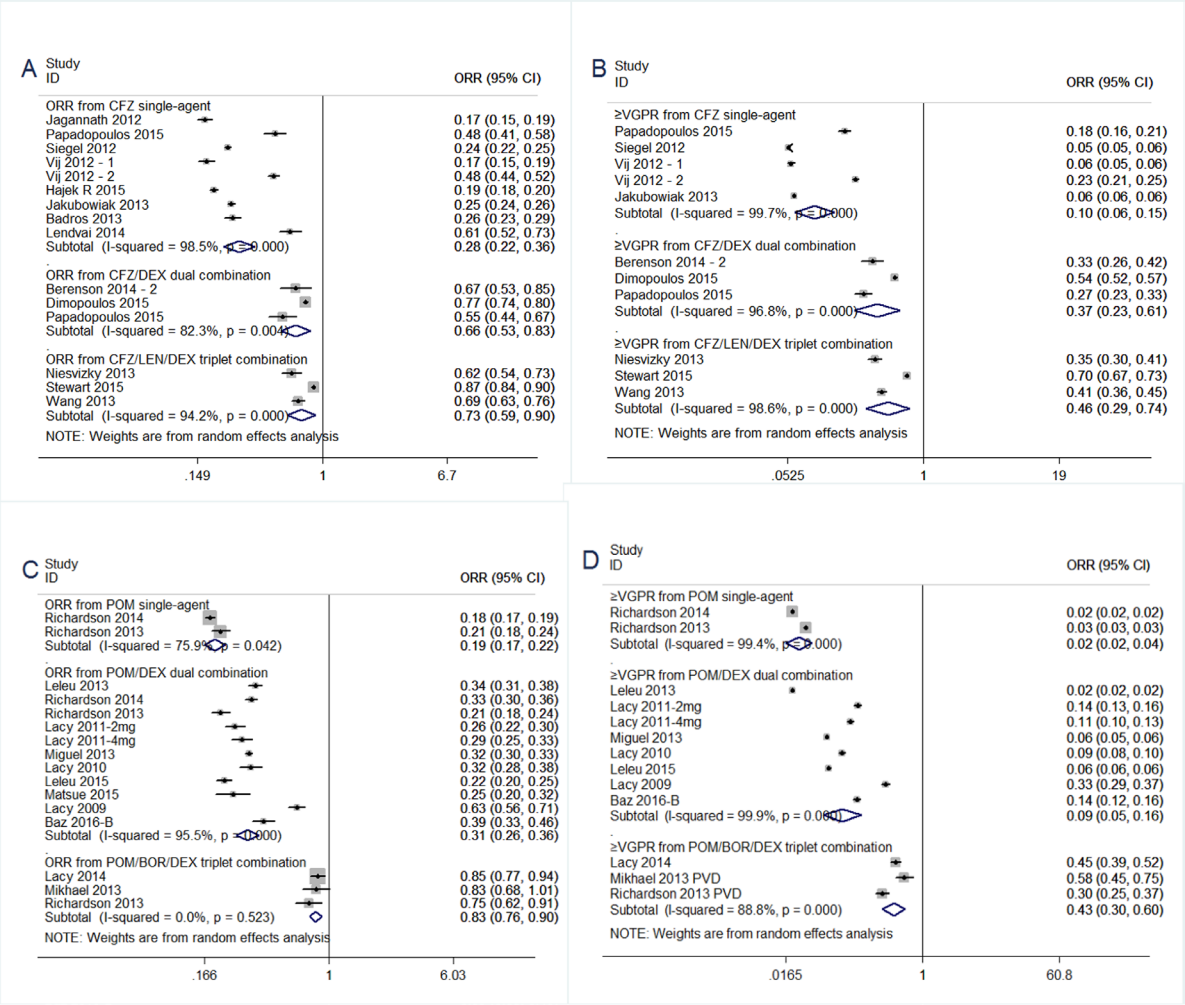
Meta-analysis of the response rate of carfilzomib/pomalidomide single agent, dual and triplet combination regimens in patients with relapsed and refractory multiple myeloma (**A**) Overall response rate of carfilzomib single-agent, CFZ/DEX dual combination, CFZ/LEN/DEX triplet combination. (**B**) At least very good partial response rate of carfilzomib single-agent, CFZ/DEX dual combination, CFZ/LEN/DEX triplet combination. (**C**) Overall response rate of pomalidomide single-agent, POM/DEX dual combination, POM/BOR/DEX triplet combination. (**D**) At least very good partial response rate of single-agent, POM/DEX dual combination, POM/BOR/DEX triplet combination. CI, 95% confidence interval. Random, random-effects model.

In order to strengthen the reliability of this pooled analysis and decrease the heterogeneity, we undertook subgoup analysis based on carfilzomib regimens (single-agent, CFZ/DEX dual combination, CFZ/LEN/DEX triplet combination), as shown in Figure 2 and Table 2. Analysis of subgroup differences between carfilzomib single-agent and CFZ/DEX dual combination showed significantly (*P* < 0.001, I^2^ = 96.3%), suggesting the overall response rate (ORR) of 66% attained from CFZ/DEX dual combination seems to be higher than that of 28% from carfilzomib single-agent. And, the same trend favoring CFZ/DEX dual combination in ≥VGPR and CBR analysis. CFZ/LEN/DEX triplet combination resulted into a similar response outcome to that from CFZ/DEX dual combination therapy in ORR, ≥VGPR, CBR and SDR analysis(Table 2). And, CFZ/POM/DEX triplet combination had a similar response outcome to that from CFZ/DEX dual combination therapy in ORR, ≥VGPR, and CBR analysis(Table 2).

### Response rate to pomalidomide single-agent and pomalidomide combination regimens

Finally, two trials enrolling a total of 146 patients evaluated the treatment effects on overall response of pomalidomide single-agent for the management of patients with RRMM. As shown in Figure 1, pooled analysis showed ORR was 19% of pomalidomide single-agent. Sixteen trials enrolling a total of 1160 patients evaluated the treatment effects on overall response of pomalidomide combination regimens in patients with RRMM. Pomalidomide combination regimens resulted into an impressive ORR of 45% (Figure 1), which was higher than that of 19% from pomalidomide single-agent (*P* < 0.001, *I*^2^ = 97.9%) (Table 2).

We undertook subgoup analysis based on pomalidomide regimens (single-agent, POM/DEX dual combination, POM/BOR/DEX triplet combination), as shown in Figure 2 and Table 2. Analysis of subgroup differences between pomalidomide single-agent and POM/DEX dual combination showed significantly (*P* < 0.001, I^2^ = 94.4%), suggesting the overall response rate (ORR) of 31% attained from POM/DEX dual combination seems to be higher than that of 19% from carfilzomib single-agent. And, the same trend favoring POM/DEX dual combination in≥VGPR and SDR analysis. POM/BOR/DEX triplet combination resulted into a superior ORR of 83% to that of 31% from POM/DEX dual combination(*P* < 0.001, *I*^2^ = 99.1%) (Table 2). And, a similar trend favoring POM/BOR/DEX triplet combination in ≥VGPR and SDR analysis.

### Adverse events

**Figure 3:**
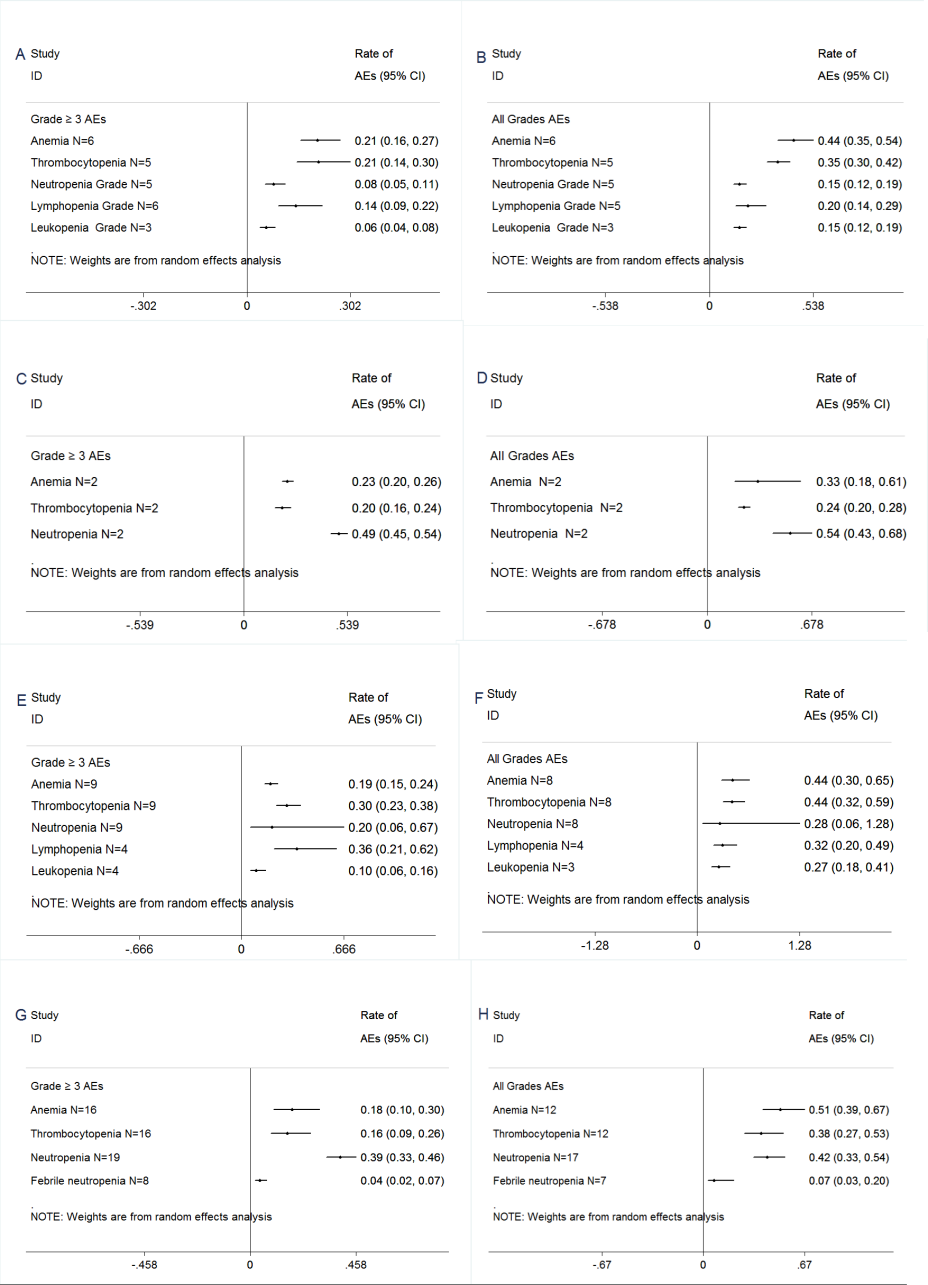
Meta-analysis of hematologic adverse events (AEs) with carfilzomib and pomalidomide for the management of patients with RRMM (**A**)≥Grade 3 hematologic AEs with carfilzomib single-agent. (**B**) All grades hematologic AEs with carfilzomib single-agent. (**C**) ≥Grade 3 hematologic AEs with pomalidomide single-agent.. (**D**) All grades hematologic AEs with pomalidomide single-agent. (**E**) ≥Grade 3 hematologic AEs with carfilzomib combinations.(**F**) All grades hematologic AEs with carfilzomib combinations.(**G**) ≥Grade 3 hematologic AEs with pomalidomide combinations.(**H**) All grades hematologic AEs with pomalidomide combinations.

**Figure 4:**
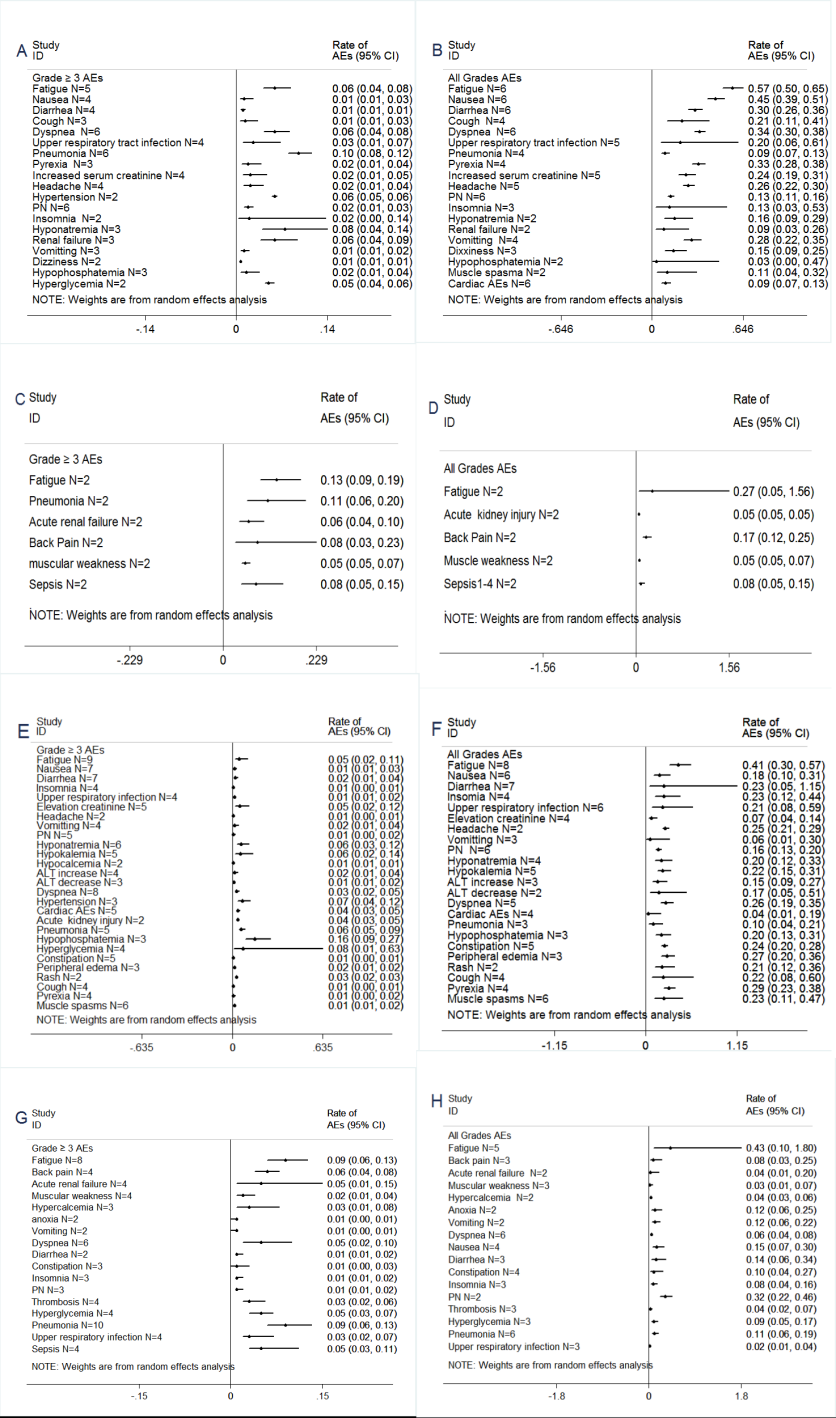
Meta-analysis of non-hematologic adverse events (AEs) with carfilzomib and pomalidomide for the management of patients with RRMM (**A**)≥Grade 3 non-hematologic AEs with carfilzomib single-agent. (**B**) All grades non-hematologic AEs with carfilzomib single-agent. (**C**) ≥Grade 3 non-hematologic AEs with pomalidomide single-agent. (**D**) All grades non-hematologic AEs with pomalidomide single-agent. (**E**) ≥Grade 3 non-hematologic AEs with carfilzomib combinations.(**F**) All grades non-hematologic AEs with carfilzomib combinations.(**G**) ≥Grade 3 non-hematologic AEs with pomalidomide combinations. (**H**) All grades non-hematologic AEs with pomalidomide combinations.

AEs were outlined in Figures 3–4. Treatment was well tolerated. In the pooled analysis, The most common AEs from carfilzomib single-agent consisted primarily of anemia (21% grade 3/4, 44% all grades), thrombocytopenia (21% grade 3/4, 35% all grades) and neutropenia (8% grade 3/4, 15% all grades), fatigue (57% all grades), nausea (45% all grades), diarrhea (30% all grades), dyspnea (34% all grades), pyrexia (33% all grades), vomitting (28% all grades) . The most common AEs from carfilzomib combination regimens consisted primarily of anemia (19% grade 3/4, 44% all grades), thrombocytopenia (30% grade 3/4, 44% all grades) and neutropenia (20% grade 3/4, 28% all grades), fatigue (41% all grades), nausea (18% all grades), diarrhea (23% all grades), dyspnea (26% all grades), pyrexia (29% all grades), vomitting (6% all grades) . Notably, there was no significant difference in these AEs analysis between carfilzomib single-agent and carfilzomib combination subgroup, except for neutropenia, nausea, vomiting.

The most common AEs from pomalidomide single-agent consisted primarily of anemia (23% grade 3/4, 33% all grades), thrombocytopenia (20% grade 3/4, 24% all grades) and neutropenia (49% grade 3/4, 54% all grades), fatigue (27% all grades), pneumonia (11% grade 3/4), acute renal failure (6% grade 3/4).

The most common AEs from pomalidomide combination regimens consisted primarily of anemia (18% grade 3/4, 51% all grades), thrombocytopenia (16% grade 3/4, 38% all grades) and neutropenia (39% grade 3/4, 42% all grades), fatigue (43% all grades), pneumonia (9% grade 3/4), acute renal failure (5% grade 3/4). Notably, there was no significant difference in these AEs analysis between pomalidomide single-agent and pomalidomide combination subgroup, except for anemia, fatigue.

## DISCUSSION

Although the proteasome inhibitor bortezomib was effective to treat myeloma patients, there were still some limits to the use of bortezomib, including occurrence of resistance and neuropathy. So, there was still a need for a second generation of proteasome inhibitors with greater efficacy and less toxicity. Carfilzomib was a potent and highly selective proteasome inhibitor, which seletively and irreversibly inhibits the chymotrypsin-like activity of the 20S proteasome. The efficacy and safety of single-agent carfilzomib had been evaluated in a series of phase 2 studies in patients with R/RMM. Because the number of patients enrolled in these trials was relatively small, we did this pooled analysis. In this aggregated analysis, the best ORR of single-agent carfilzomib for the response evaluable population was 28.0% and the CBR was 37.0%. These results reinforce the efficacy with carfilzomib monotherapy for a significant number of heavily pretreated patients. The minimal off-target activity characteristic and minimal neurotoxicity of carfilzomib supported it's use in combination with other agent. Pooling three trials of CFZ/DEX dual combination regimen in the 501 relapsed/refractory patients, the 66% ORR attained with CFZ/DEX dual combination regimen was impressive, particularly when considering the 28% ORR achieved with single-agent carfilzomib in a similar population with R/RMM. Furthermore, there was no significant difference between the CFZ/DEX dual and CFZ/LEN/DEX triplet combination in ORR,≥VGPR,CBR,SDR analysis. So, CFZ/DEX dual regimen still should be good option for patients with RRMM.

Carfilzomib single agent was generally tolerable, with the majority of patients receiving the planned dose. The most common treatment-related AEs were gastrointestinal (nausea, diarrhoea, vomiting, and constipation), fatigue, dyspnea, and myelosuppression (thrombocytopenia, anaemia, and neutropenia). Peripheral neuropathy(PN) was reported infrequently. Comparison of tolerability between carfilzomib and bortezomib containing combination regimens should also be made with caution, but the difference in peripheral neuropathy was notable. Rates of grade ≥ 2 PN were 6.3% from CFZ/DEX dual combination *vs* 32.0% from POM/DEX dual combination(*P* < .0001) in ENDEAVOR trial [39]. The result was very encouraging, because PN was the main reason leading to discontinuation of bortezomib.

Preclinical study had shown that the efficacy of pomalidomide might be enhanced by the addition of dexamethasone. This current study pooled from 9 prospective trials of refractory multiple myeloma who received pomalidomide dual combination (POM+LoDEX) after failure of lenalidomide and bortezomib therapy, and strengthened the individual observations of each of these small prospective studies alone. The ORRs of 31% with POM+LoDEX was impressive, which compared favorably with 19% in the POM alone arm (Figure 2, Table 2), and 10% in the DEX alone [46], in consistent with the synergistic action of POM+LoDEX as observed in previous *in vitro* studies [47].And, the States Food and Drug Administration already approved pomalidomide in combination with dexamethasone in February2013 for patients with relapsed and refractory multiple myeloma. Recently, there were several trials of pomalidomide triplet combinations. When pooling three trials of POM/BOR/DEX triplet combination, an high ORR of 83% was achieved, which was superior to that of 31% (Table 2). This finding was impressive, and needed further validation in future trials.

When interpreting our results, there were some caveats that should be considered. The first and major problem was that we used abstracted data, whereas an individual patient data-based meta-analysis might define more clearly treatment efficacy of carfilzomib and pomalidomide. Secondly, as was often the case with meta-analysis, the effect of heterogeneity needed to be taken into account. Although all studies were discussed about the objective response of carfizlomib and pomalidomide after disease progression on lenalidomide and (or) bortezomib, the inclusion criteria were different among individual studies.

Pomalidomide/Carfilzomib plus dexamethasone seemed to attain a superior response rate compared with pomalidomide/carfilzomib single-agent. Furthermore, the combination of pomalidomide, bortezomib and dexamethasone resulted in a much higher response rate compared with pomalidomide plus dexamethasone regimen. These results required validation in future.

## MATERIALS AND METHODS

### Literature search strategy

Medline, Embase, the Cochrane controlled trials register, the Science Citation Index, Conference proceedings from the American Society of Hematology(ASH), the European Hematology association (EHA) and the American Society of Clinical Oncology were searched for trials using the medical subject headings “myeloma”, “carfilzomib”, “pomalidomide”, “bortezomib” and “lenalidomide”. Reference lists from studies selected for this review, and from other published systematic reviews and practice guidelines were also hand-searched. The study was approved by the institutional review boards of Weifang People's Hospital,in accordance with the Helsinki Declaration.

### Selection of studies

Studies were eligible for inclusion in the meta-analysis if they met all the following criteria: (1) They were published up to February, 2016 and written in English. (2) They dealt only with patients with refractory or relapsed multiple myeloma who had received bortezomib and (or) lenalidomide. (3) Study selection included the setting of these trials: carfilzomib/pomalidomide single-agent, dual and triplet combination regimens. (4) We included studies that provided sufficient information to allow the calculation of response rate. Multiple reports of a single study were considered as one publication, and only the most recent or complete article was examined. All potentially relevant articles were reviewed by two independent investigators (X.H.R and Y.D.Z.).

### Outcome measures

The primary objective of the study was to determine the overall response rate (ORR = ≥PR), at least very good partial response (VGPR), clinical benefit rate (CBR = ≥MR), stable disease rate (SDR), progressive disease rate (PDR) of pomalidomide dual and triplet combination regimens, and the secondary objectives were to evaluate the safety of pomalidomide combinations in this population. Responses were investigator assessed based on modified European Group for Bone Marrow Transplantation criteria [42, 43] and International Myeloma Working Group uniform response criteria [44]. National Cancer Institute Common Toxicity Criteria (NCICTC) was used to grade adverse events (AEs).

### Statistical analysis

A random-effects model was used for all the analyses, which incorporates the variability of results among trials and provided a more conservative estimate of an effect size by producing greater confidence intervals (CIs) [45]. We tested for heterogeneity of between-study with the Cochrane χ^2^ test and quantified its extent with the *I*^2^ statistic. If significant heterogeneity existed, it would be appropriate to pool the data using random-effects model, but not fixed-effect model. All meta-analyses were completed using Stata ver. 12.0 software (College Station, TX) and Review Manager (version 5.3; Th e Cochrane Collaboration, Oxford, England). Statistical significance was defined as a P value of less than 0.05 for all tests.
